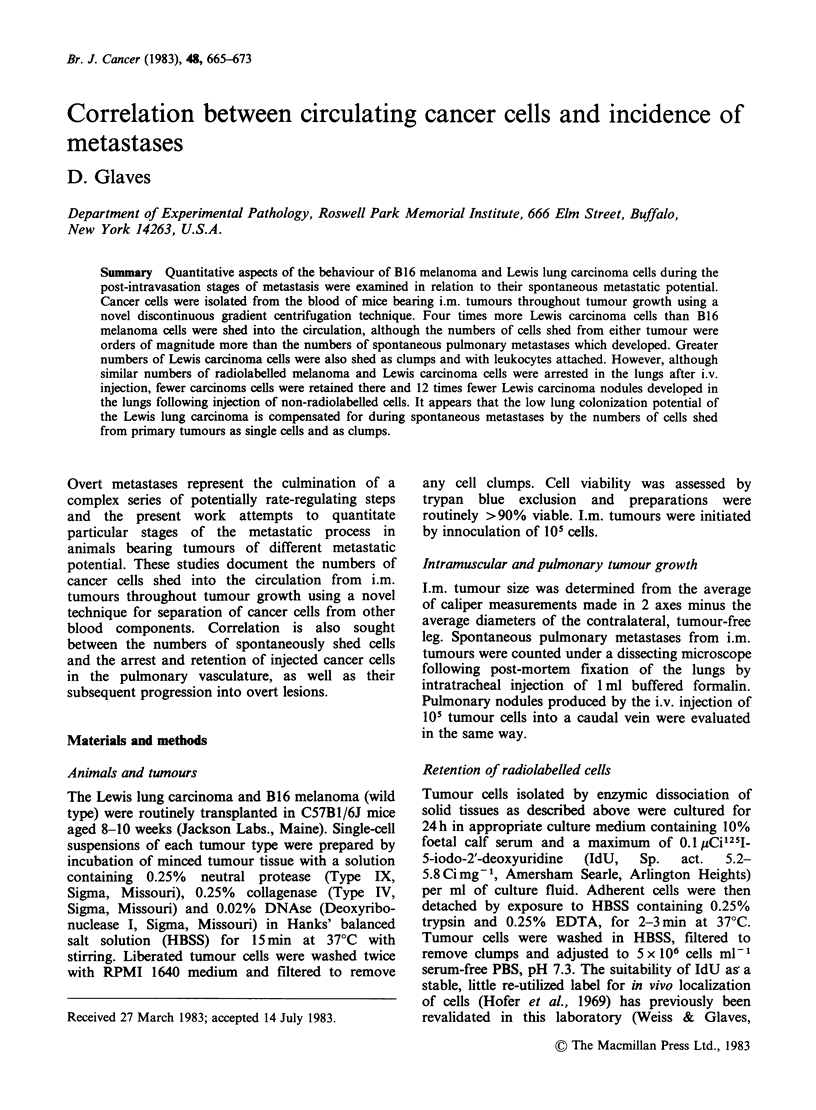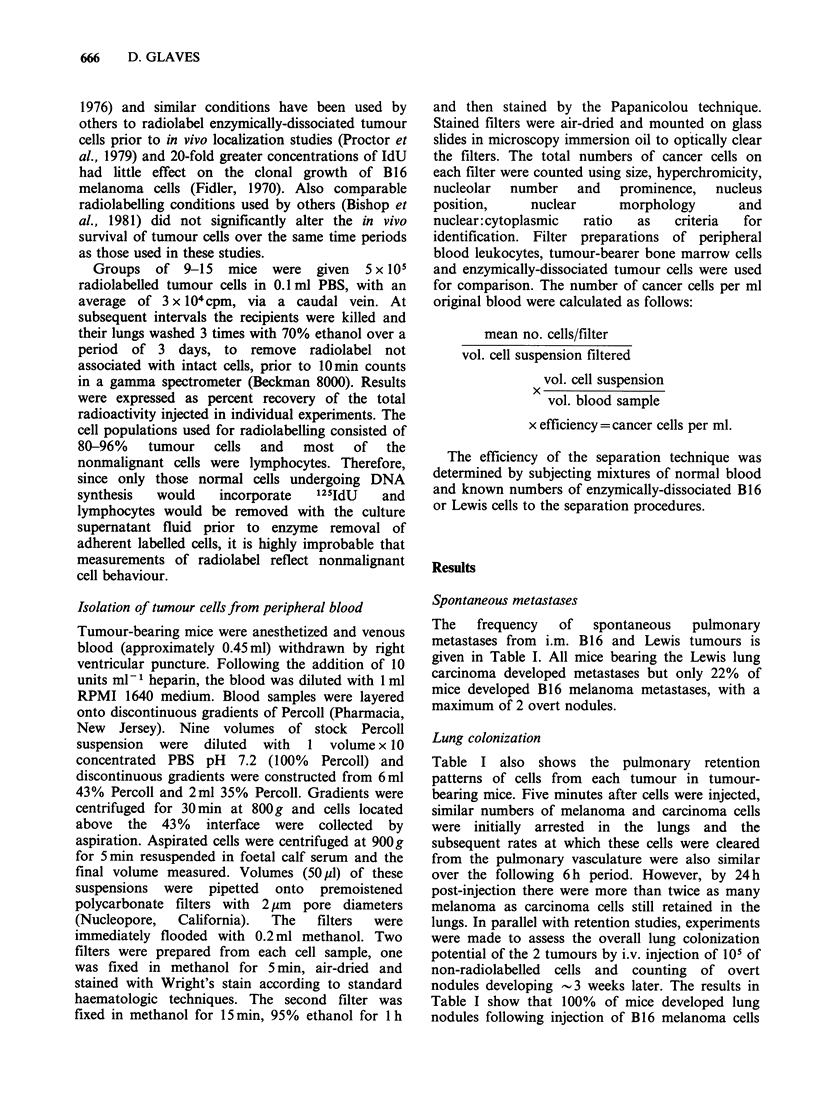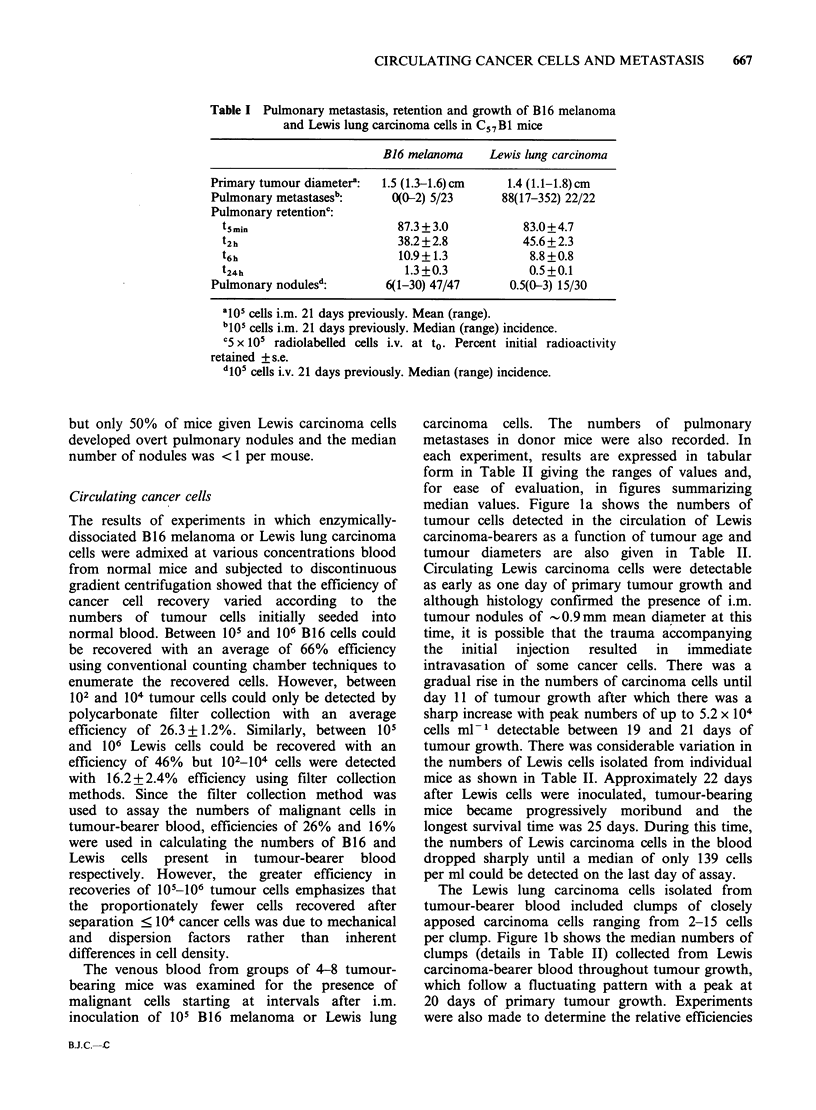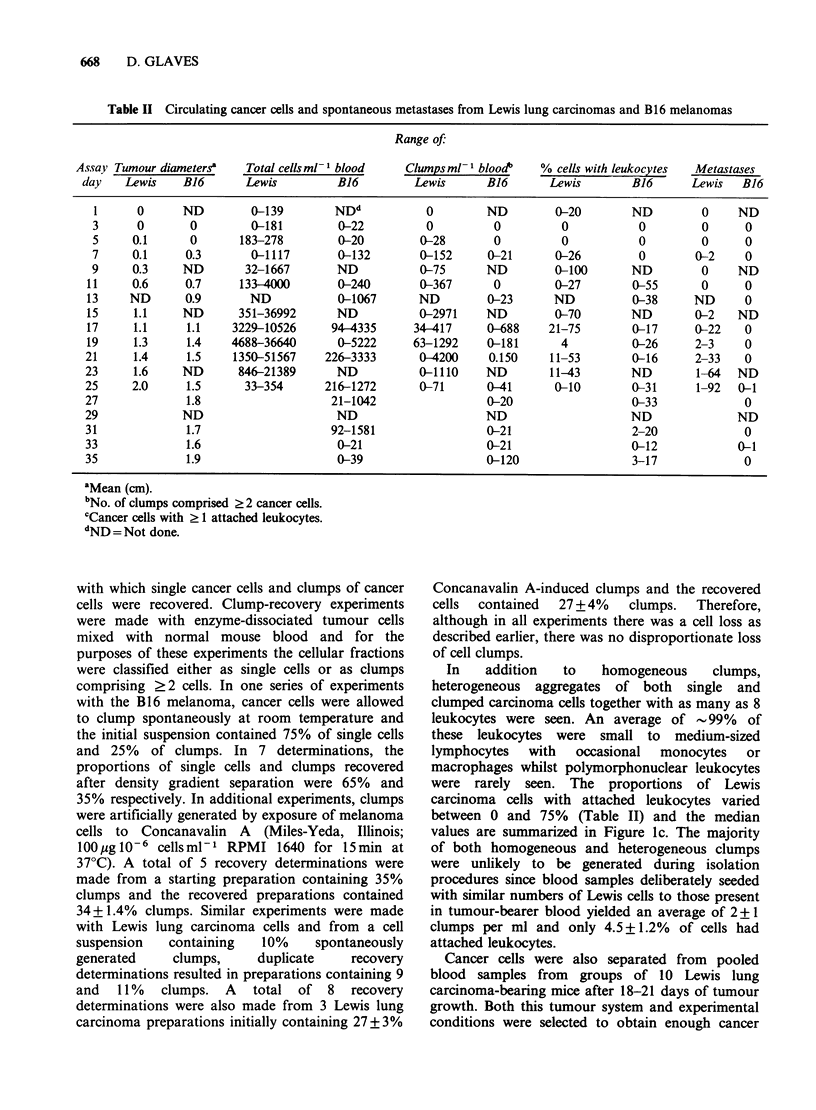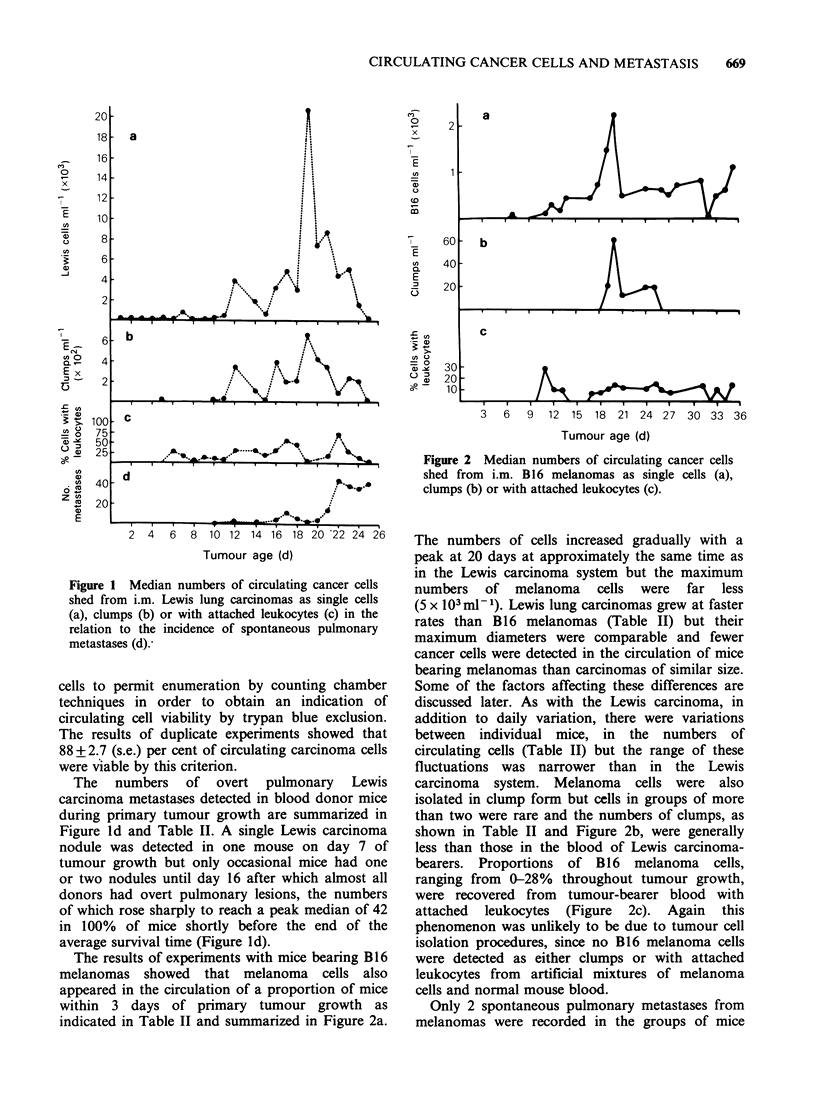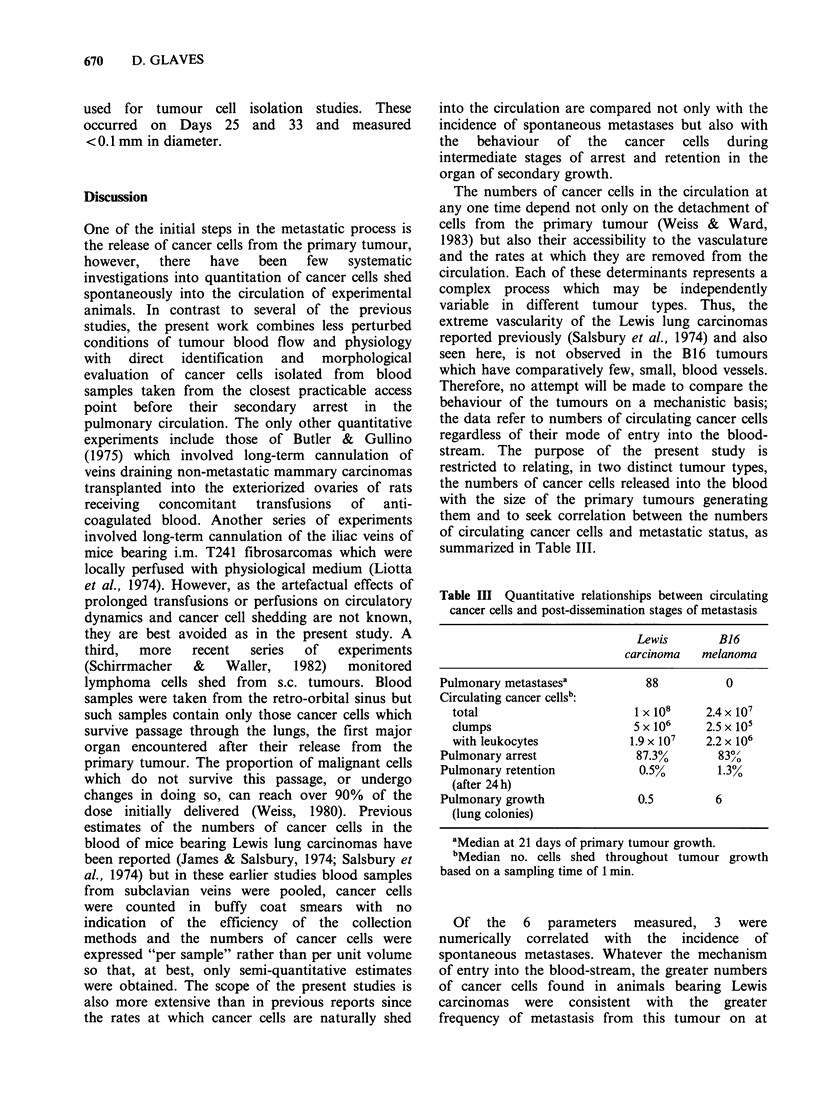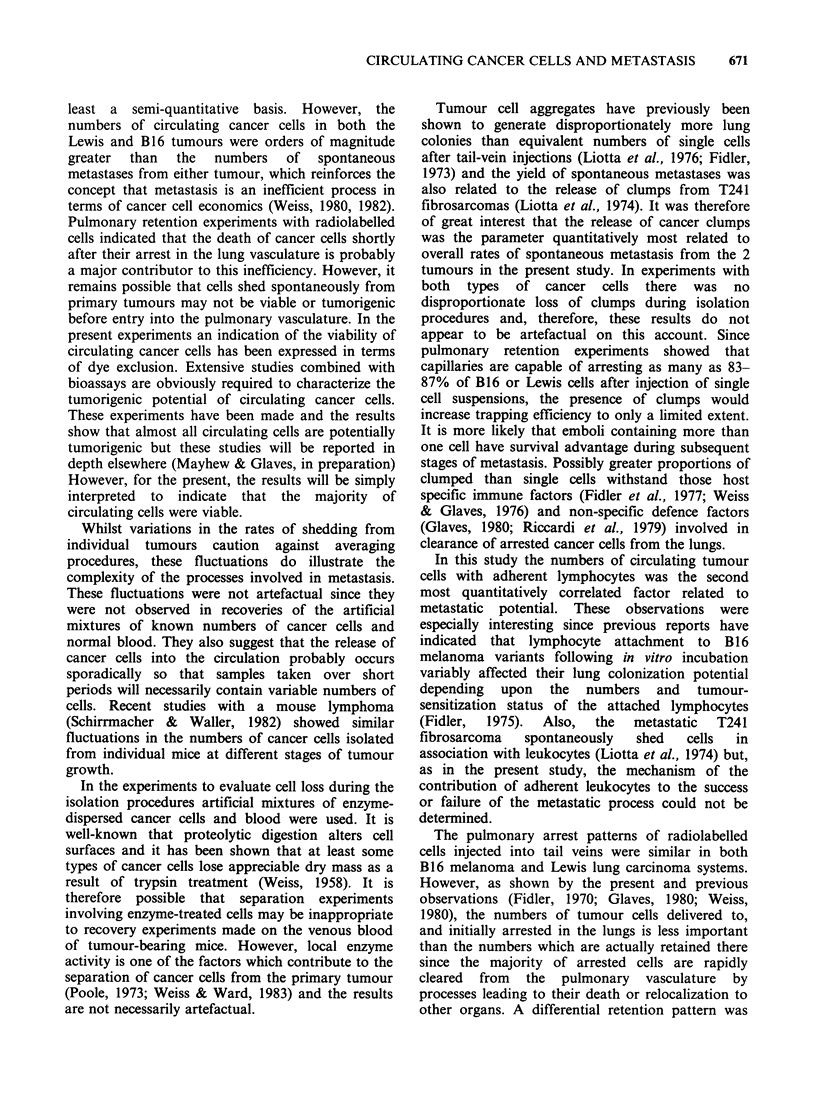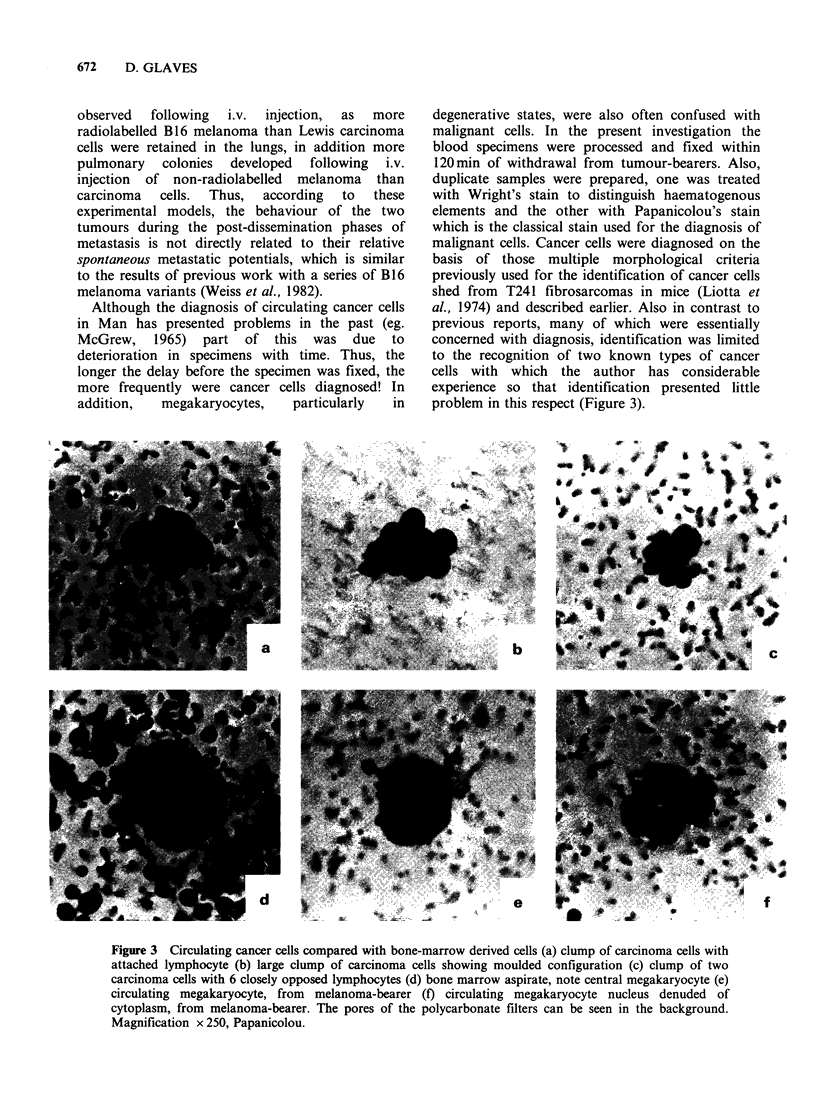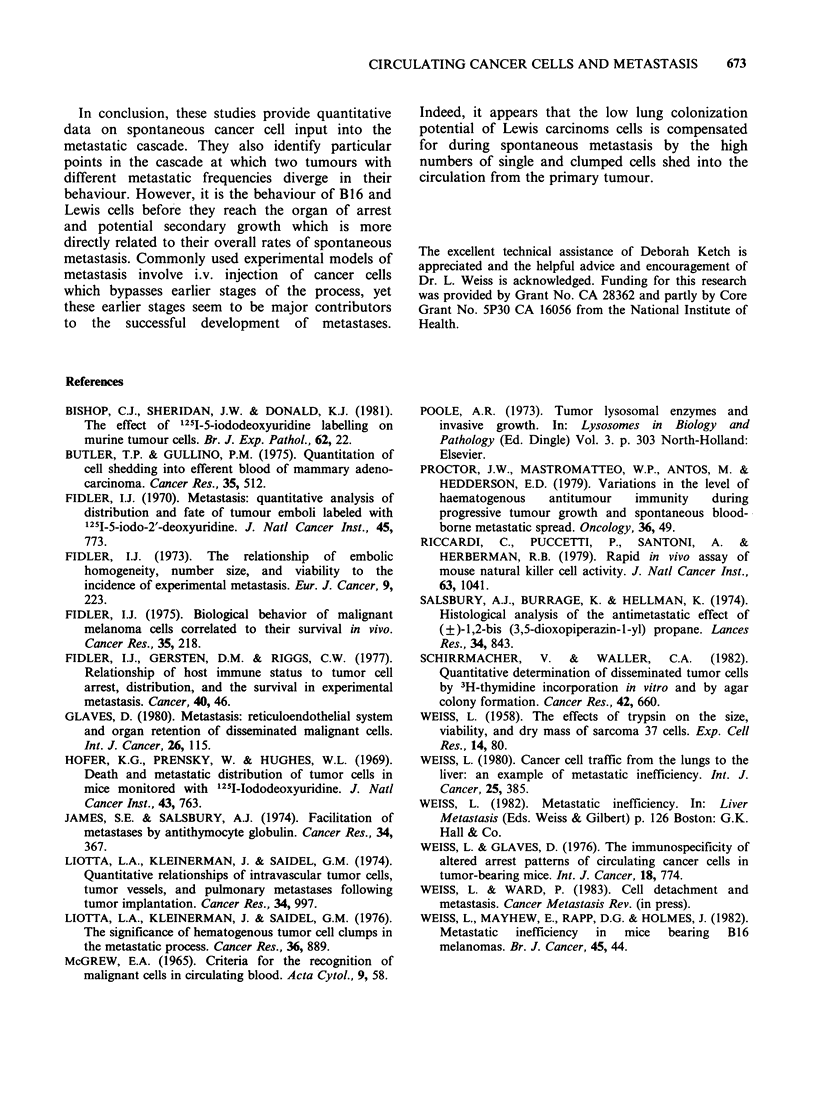# Correlation between circulating cancer cells and incidence of metastases.

**DOI:** 10.1038/bjc.1983.248

**Published:** 1983-11

**Authors:** D. Glaves

## Abstract

**Images:**


*Br. J. Cancer* (1983), **48**, 665–673

### Correlation between circulating cancer cells and incidence of metastases

D. Glaves

*Department of Experimental Pathology, Roswell Park Memorial Institute, 666 Elm Street, Buffalo, New York 14263, U.S.A.*

**Summary** Quantitative aspects of the behaviour of B16 melanoma and Lewis lung carcinoma cells during the post-intravasation stages of metastasis were examined in relation to their spontaneous metastatic potential. Cancer cells were isolated from the blood of mice bearing i.m. tumours throughout tumour growth using a novel discontinuous gradient centrifugation technique. Four times more Lewis carcinoma cells than B16 melanoma cells were shed into the circulation, although the numbers of cells shed from either tumour were orders of magnitude more than the numbers of spontaneous pulmonary metastases which developed. Greater numbers of Lewis carcinoma cells were also shed as clumps and with leukocytes attached. However, although similar numbers of radiolabelled melanoma and Lewis carcinoma cells were arrested in the lungs after i.v. injection, fewer carcinoms cells were retained there and 12 times fewer Lewis carcinoma nodules developed in the lungs following injection of non-radiolabelled cells. It appears that the low lung colonization potential of the Lewis lung carcinoma is compensated for during spontaneous metastases by the numbers of cells shed from primary tumours as single cells and as clumps.

Overt metastases represent the culmination of a complex series of potentially rate-regulating steps and the present work attempts to quantitate particular stages of the metastatic process in animals bearing tumours of different metastatic potential. These studies document the numbers of cancer cells shed into the circulation from i.m. tumours throughout tumour growth using a novel technique for separation of cancer cells from other blood components. Correlation is also sought between the numbers of spontaneously shed cells and the arrest and retention of injected cancer cells in the pulmonary vasculature, as well as their subsequent progression into overt lesions.

### Materials and methods

### *Animals and tumours*

The Lewis lung carcinoma and B16 melanoma (wild type) were routinely transplanted in C57Bl/6J mice aged 8–10 weeks (Jackson Labs., Maine). Single-cell suspensions of each tumour type were prepared by incubation of minced tumour tissue with a solution containing 0.25% neutral protease (Type IX, Sigma, Missouri), 0.25% collagenase (Type IV, Sigma, Missouri) and 0.02% DNAse (Deoxyribonuclease I, Sigma, Missouri) in Hanks' balanced salt solution (HBSS) for 15 min at 37°C with stirring. Liberated tumour cells were washed twice with RPMI 1640 medium and filtered to remove any cell clumps. Cell viability was assessed by trypan blue exclusion and preparations were routinely >90% viable. I.m. tumours were initiated by innoculation of $10^5$ cells.

Received 27 March 1983; accepted 14 July 1983.

### *Intramuscular and pulmonary tumour growth*

I.m. tumour size was determined from the average of caliper measurements made in 2 axes minus the average diameters of the contralateral, tumour-free leg. Spontaneous pulmonary metastases from i.m. tumours were counted under a dissecting microscope following post-mortem fixation of the lungs by intratracheal injection of 1 ml buffered formalin. Pulmonary nodules produced by the i.v. injection of $10^5$ tumour cells into a caudal vein were evaluated in the same way.

### *Retention of radiolabelled cells*

Tumour cells isolated by enzymic dissociation of solid tissues as described above were cultured for 24 h in appropriate culture medium containing 10% foetal calf serum and a maximum of 0.1 $\mu$Ci $^{125}$I-5-iodo-2′-deoxyuridine (IdU, Sp. act. 5.2–5.8 Ci mg$^{-1}$, Amersham Searle, Arlington Heights) per ml of culture fluid. Adherent cells were then detached by exposure to HBSS containing 0.25% trypsin and 0.25% EDTA, for 2–3 min at 37°C. Tumour cells were washed in HBSS, filtered to remove clumps and adjusted to $5 \times 10^6$ cells ml$^{-1}$ serum-free PBS, pH 7.3. The suitability of IdU as a stable, little re-utilized label for *in vivo* localization of cells (Hofer *et al.*, 1969) has previously been revalidated in this laboratory (Weiss & Glaves,

© The Macmillan Press Ltd., 1983

1976) and similar conditions have been used by others to radiolabel enzymically-dissociated tumour cells prior to *in vivo* localization studies (Proctor *et al.*, 1979) and 20-fold greater concentrations of IdU had little effect on the clonal growth of B16 melanoma cells (Fidler, 1970). Also comparable radiolabelling conditions used by others (Bishop *et al.*, 1981) did not significantly alter the *in vivo* survival of tumour cells over the same time periods as those used in these studies.

Groups of 9–15 mice were given $5 \times 10^5$ radiolabelled tumour cells in 0.1 ml PBS, with an average of $3 \times 10^4$ cpm, via a caudal vein. At subsequent intervals the recipients were killed and their lungs washed 3 times with 70% ethanol over a period of 3 days, to remove radiolabel not associated with intact cells, prior to 10 min counts in a gamma spectrometer (Beckman 8000). Results were expressed as percent recovery of the total radioactivity injected in individual experiments. The cell populations used for radiolabelling consisted of 80–96% tumour cells and most of the nonmalignant cells were lymphocytes. Therefore, since only those normal cells undergoing DNA synthesis would incorporate $^{125}$IdU and lymphocytes would be removed with the culture supernatant fluid prior to enzyme removal of adherent labelled cells, it is highly improbable that measurements of radiolabel reflect nonmalignant cell behaviour.

*Isolation of tumour cells from peripheral blood*

Tumour-bearing mice were anesthetized and venous blood (approximately 0.45 ml) withdrawn by right ventricular puncture. Following the addition of 10 units ml$^{-1}$ heparin, the blood was diluted with 1 ml RPMI 1640 medium. Blood samples were layered onto discontinuous gradients of Percoll (Pharmacia, New Jersey). Nine volumes of stock Percoll suspension were diluted with 1 volume × 10 concentrated PBS pH 7.2 (100% Percoll) and discontinuous gradients were constructed from 6 ml 43% Percoll and 2 ml 35% Percoll. Gradients were centrifuged for 30 min at 800 *g* and cells located above the 43% interface were collected by aspiration. Aspirated cells were centrifuged at 900 *g* for 5 min resuspended in foetal calf serum and the final volume measured. Volumes (50 $\mu$l) of these suspensions were pipetted onto premoistened polycarbonate filters with 2 $\mu$m pore diameters (Nucleopore, California). The filters were immediately flooded with 0.2 ml methanol. Two filters were prepared from each cell sample, one was fixed in methanol for 5 min, air-dried and stained with Wright's stain according to standard haematologic techniques. The second filter was fixed in methanol for 15 min, 95% ethanol for 1 h and then stained by the Papanicolou technique. Stained filters were air-dried and mounted on glass slides in microscopy immersion oil to optically clear the filters. The total numbers of cancer cells on each filter were counted using size, hyperchromicity, nucleolar number and prominence, nucleus position, nuclear morphology and nuclear:cytoplasmic ratio as criteria for identification. Filter preparations of peripheral blood leukocytes, tumour-bearer bone marrow cells and enzymically-dissociated tumour cells were used for comparison. The number of cancer cells per ml original blood were calculated as follows:

$$\frac{\text{mean no. cells/filter}}{\text{vol. cell suspension filtered}} \times \frac{\text{vol. cell suspension}}{\text{vol. blood sample}} \times \text{efficiency} = \text{cancer cells per ml.}$$

The efficiency of the separation technique was determined by subjecting mixtures of normal blood and known numbers of enzymically-dissociated B16 or Lewis cells to the separation procedures.

### Results

*Spontaneous metastases*

The frequency of spontaneous pulmonary metastases from i.m. B16 and Lewis tumours is given in Table I. All mice bearing the Lewis lung carcinoma developed metastases but only 22% of mice developed B16 melanoma metastases, with a maximum of 2 overt nodules.

*Lung colonization*

Table I also shows the pulmonary retention patterns of cells from each tumour in tumour-bearing mice. Five minutes after cells were injected, similar numbers of melanoma and carcinoma cells were initially arrested in the lungs and the subsequent rates at which these cells were cleared from the pulmonary vasculature were also similar over the following 6 h period. However, by 24 h post-injection there were more than twice as many melanoma as carcinoma cells still retained in the lungs. In parallel with retention studies, experiments were made to assess the overall lung colonization potential of the 2 tumours by i.v. injection of $10^5$ of non-radiolabelled cells and counting of overt nodules developing ~3 weeks later. The results in Table I show that 100% of mice developed lung nodules following injection of B16 melanoma cells

**Table I** Pulmonary metastasis, retention and growth of B16 melanoma and Lewis lung carcinoma cells in $C_{57}Bl$ mice

| | *B16 melanoma* | *Lewis lung carcinoma* |
|---|---|---|
| Primary tumour diameter[a]: | 1.5 (1.3–1.6) cm | 1.4 (1.1–1.8) cm |
| Pulmonary metastases[b]: | 0(0–2) 5/23 | 88(17–352) 22/22 |
| Pulmonary retention[c]: | | |
| $t_{5\,min}$ | 87.3±3.0 | 83.0±4.7 |
| $t_{2h}$ | 38.2±2.8 | 45.6±2.3 |
| $t_{6h}$ | 10.9±1.3 | 8.8±0.8 |
| $t_{24h}$ | 1.3±0.3 | 0.5±0.1 |
| Pulmonary nodules[d]: | 6(1–30) 47/47 | 0.5(0–3) 15/30 |

[a]$10^5$ cells i.m. 21 days previously. Mean (range).
[b]$10^5$ cells i.m. 21 days previously. Median (range) incidence.
[c]$5\times10^5$ radiolabelled cells i.v. at $t_0$. Percent initial radioactivity retained ±s.e.
[d]$10^5$ cells i.v. 21 days previously. Median (range) incidence.

but only 50% of mice given Lewis carcinoma cells developed overt pulmonary nodules and the median number of nodules was <1 per mouse.

*Circulating cancer cells*

The results of experiments in which enzymically-dissociated B16 melanoma or Lewis lung carcinoma cells were admixed at various concentrations blood from normal mice and subjected to discontinuous gradient centrifugation showed that the efficiency of cancer cell recovery varied according to the numbers of tumour cells initially seeded into normal blood. Between $10^5$ and $10^6$ B16 cells could be recovered with an average of 66% efficiency using conventional counting chamber techniques to enumerate the recovered cells. However, between $10^2$ and $10^4$ tumour cells could only be detected by polycarbonate filter collection with an average efficiency of 26.3±1.2%. Similarly, between $10^5$ and $10^6$ Lewis cells could be recovered with an efficiency of 46% but $10^2$–$10^4$ cells were detected with 16.2±2.4% efficiency using filter collection methods. Since the filter collection method was used to assay the numbers of malignant cells in tumour-bearer blood, efficiencies of 26% and 16% were used in calculating the numbers of B16 and Lewis cells present in tumour-bearer blood respectively. However, the greater efficiency in recoveries of $10^5$–$10^6$ tumour cells emphasizes that the proportionately fewer cells recovered after separation $\leq10^4$ cancer cells was due to mechanical and dispersion factors rather than inherent differences in cell density.

The venous blood from groups of 4–8 tumour-bearing mice was examined for the presence of malignant cells starting at intervals after i.m. inoculation of $10^5$ B16 melanoma or Lewis lung carcinoma cells. The numbers of pulmonary metastases in donor mice were also recorded. In each experiment, results are expressed in tabular form in Table II giving the ranges of values and, for ease of evaluation, in figures summarizing median values. Figure 1a shows the numbers of tumour cells detected in the circulation of Lewis carcinoma-bearers as a function of tumour age and tumour diameters are also given in Table II. Circulating Lewis carcinoma cells were detectable as early as one day of primary tumour growth and although histology confirmed the presence of i.m. tumour nodules of ~0.9 mm mean diameter at this time, it is possible that the trauma accompanying the initial injection resulted in immediate intravasation of some cancer cells. There was a gradual rise in the numbers of carcinoma cells until day 11 of tumour growth after which there was a sharp increase with peak numbers of up to $5.2\times10^4$ cells $ml^{-1}$ detectable between 19 and 21 days of tumour growth. There was considerable variation in the numbers of Lewis cells isolated from individual mice as shown in Table II. Approximately 22 days after Lewis cells were inoculated, tumour-bearing mice became progressively moribund and the longest survival time was 25 days. During this time, the numbers of Lewis carcinoma cells in the blood dropped sharply until a median of only 139 cells per ml could be detected on the last day of assay.

The Lewis lung carcinoma cells isolated from tumour-bearer blood included clumps of closely apposed carcinoma cells ranging from 2–15 cells per clump. Figure 1b shows the median numbers of clumps (details in Table II) collected from Lewis carcinoma-bearer blood throughout tumour growth, which follow a fluctuating pattern with a peak at 20 days of primary tumour growth. Experiments were also made to determine the relative efficiencies

**Table II** Circulating cancer cells and spontaneous metastases from Lewis lung carcinomas and B16 melanomas

| Assay day | Tumour diameters[a] | | Total cells $ml^{-1}$ blood | | Clumps $ml^{-1}$ blood[b] | | % cells with leukocytes | | Metastases | |
|---|---|---|---|---|---|---|---|---|---|---|
| | *Lewis* | *B16* | *Lewis* | *B16* | *Lewis* | *B16* | *Lewis* | *B16* | *Lewis* | *B16* |
| 1 | 0 | ND | 0–139 | ND[d] | 0 | ND | 0–20 | ND | 0 | ND |
| 3 | 0 | 0 | 0–181 | 0–22 | 0 | 0 | 0 | 0 | 0 | 0 |
| 5 | 0.1 | 0 | 183–278 | 0–20 | 0–28 | 0 | 0 | 0 | 0 | 0 |
| 7 | 0.1 | 0.3 | 0–1117 | 0–132 | 0–152 | 0–21 | 0–26 | 0 | 0–2 | 0 |
| 9 | 0.3 | ND | 32–1667 | ND | 0–75 | ND | 0–100 | ND | 0 | ND |
| 11 | 0.6 | 0.7 | 133–4000 | 0–240 | 0–367 | 0 | 0–27 | 0–55 | 0 | 0 |
| 13 | ND | 0.9 | ND | 0–1067 | ND | 0–23 | ND | 0–38 | ND | 0 |
| 15 | 1.1 | ND | 351–36992 | ND | 0–2971 | ND | 0–70 | ND | 0–2 | ND |
| 17 | 1.1 | 1.1 | 3229–10526 | 94–4335 | 34–417 | 0–688 | 21–75 | 0–17 | 0–22 | 0 |
| 19 | 1.3 | 1.4 | 4688–36640 | 0–5222 | 63–1292 | 0–181 | 4 | 0–26 | 2–3 | 0 |
| 21 | 1.4 | 1.5 | 1350–51567 | 226–3333 | 0–4200 | 0.150 | 11–53 | 0–16 | 2–33 | 0 |
| 23 | 1.6 | ND | 846–21389 | ND | 0–1110 | ND | 11–43 | ND | 1–64 | ND |
| 25 | 2.0 | 1.5 | 33–354 | 216–1272 | 0–71 | 0–41 | 0–10 | 0–31 | 1–92 | 0–1 |
| 27 | | 1.8 | | 21–1042 | | 0–20 | | 0–33 | | 0 |
| 29 | | ND | | ND | | ND | | ND | | ND |
| 31 | | 1.7 | | 92–1581 | | 0–21 | | 2–20 | | 0 |
| 33 | | 1.6 | | 0–21 | | 0–21 | | 0–12 | | 0–1 |
| 35 | | 1.9 | | 0–39 | | 0–120 | | 3–17 | | 0 |

Range of:

[a]Mean (cm).
[b]No. of clumps comprised ≥2 cancer cells.
[c]Cancer cells with ≥1 attached leukocytes.
[d]ND = Not done.

with which single cancer cells and clumps of cancer cells were recovered. Clump-recovery experiments were made with enzyme-dissociated tumour cells mixed with normal mouse blood and for the purposes of these experiments the cellular fractions were classified either as single cells or as clumps comprising ≥2 cells. In one series of experiments with the B16 melanoma, cancer cells were allowed to clump spontaneously at room temperature and the initial suspension contained 75% of single cells and 25% of clumps. In 7 determinations, the proportions of single cells and clumps recovered after density gradient separation were 65% and 35% respectively. In additional experiments, clumps were artificially generated by exposure of melanoma cells to Concanavalin A (Miles-Yeda, Illinois; $100 \mu g\ 10^{-6}$ cells $ml^{-1}$ RPMI 1640 for 15 min at 37°C). A total of 5 recovery determinations were made from a starting preparation containing 35% clumps and the recovered preparations contained 34±1.4% clumps. Similar experiments were made with Lewis lung carcinoma cells and from a cell suspension containing 10% spontaneously generated clumps, duplicate recovery determinations resulted in preparations containing 9 and 11% clumps. A total of 8 recovery determinations were also made from 3 Lewis lung carcinoma preparations initially containing 27±3% Concanavalin A-induced clumps and the recovered cells contained 27±4% clumps. Therefore, although in all experiments there was a cell loss as described earlier, there was no disproportionate loss of cell clumps.

In addition to homogeneous clumps, heterogeneous aggregates of both single and clumped carcinoma cells together with as many as 8 leukocytes were seen. An average of ~99% of these leukocytes were small to medium-sized lymphocytes with occasional monocytes or macrophages whilst polymorphonuclear leukocytes were rarely seen. The proportions of Lewis carcinoma cells with attached leukocytes varied between 0 and 75% (Table II) and the median values are summarized in Figure 1c. The majority of both homogeneous and heterogeneous clumps were unlikely to be generated during isolation procedures since blood samples deliberately seeded with similar numbers of Lewis cells to those present in tumour-bearer blood yielded an average of 2±1 clumps per ml and only 4.5±1.2% of cells had attached leukocytes.

Cancer cells were also separated from pooled blood samples from groups of 10 Lewis lung carcinoma-bearing mice after 18–21 days of tumour growth. Both this tumour system and experimental conditions were selected to obtain enough cancer

**Figure 1** Median numbers of circulating cancer cells shed from i.m. Lewis lung carcinomas as single cells (a), clumps (b) or with attached leukocytes (c) in the relation to the incidence of spontaneous pulmonary metastases (d).

**Figure 2** Median numbers of circulating cancer cells shed from i.m. B16 melanomas as single cells (a), clumps (b) or with attached leukocytes (c).

cells to permit enumeration by counting chamber techniques in order to obtain an indication of circulating cell viability by trypan blue exclusion. The results of duplicate experiments showed that $88 \pm 2.7$ (s.e.) per cent of circulating carcinoma cells were viable by this criterion.

The numbers of overt pulmonary Lewis carcinoma metastases detected in blood donor mice during primary tumour growth are summarized in Figure 1d and Table II. A single Lewis carcinoma nodule was detected in one mouse on day 7 of tumour growth but only occasional mice had one or two nodules until day 16 after which almost all donors had overt pulmonary lesions, the numbers of which rose sharply to reach a peak median of 42 in 100% of mice shortly before the end of the average survival time (Figure 1d).

The results of experiments with mice bearing B16 melanomas showed that melanoma cells also appeared in the circulation of a proportion of mice within 3 days of primary tumour growth as indicated in Table II and summarized in Figure 2a. The numbers of cells increased gradually with a peak at 20 days at approximately the same time as in the Lewis carcinoma system but the maximum numbers of melanoma cells were far less ($5 \times 10^3\,ml^{-1}$). Lewis lung carcinomas grew at faster rates than B16 melanomas (Table II) but their maximum diameters were comparable and fewer cancer cells were detected in the circulation of mice bearing melanomas than carcinomas of similar size. Some of the factors affecting these differences are discussed later. As with the Lewis carcinoma, in addition to daily variation, there were variations between individual mice, in the numbers of circulating cells (Table II) but the range of these fluctuations was narrower than in the Lewis carcinoma system. Melanoma cells were also isolated in clump form but cells in groups of more than two were rare and the numbers of clumps, as shown in Table II and Figure 2b, were generally less than those in the blood of Lewis carcinoma-bearers. Proportions of B16 melanoma cells, ranging from 0–28% throughout tumour growth, were recovered from tumour-bearer blood with attached leukocytes (Figure 2c). Again this phenomenon was unlikely to be due to tumour cell isolation procedures, since no B16 melanoma cells were detected as either clumps or with attached leukocytes from artificial mixtures of melanoma cells and normal mouse blood.

Only 2 spontaneous pulmonary metastases from melanomas were recorded in the groups of mice used for tumour cell isolation studies. These occurred on Days 25 and 33 and measured <0.1 mm in diameter.

### Discussion

One of the initial steps in the metastatic process is the release of cancer cells from the primary tumour, however, there have been few systematic investigations into quantitation of cancer cells shed spontaneously into the circulation of experimental animals. In contrast to several of the previous studies, the present work combines less perturbed conditions of tumour blood flow and physiology with direct identification and morphological evaluation of cancer cells isolated from blood samples taken from the closest practicable access point before their secondary arrest in the pulmonary circulation. The only other quantitative experiments include those of Butler & Gullino (1975) which involved long-term cannulation of veins draining non-metastatic mammary carcinomas transplanted into the exteriorized ovaries of rats receiving concomitant transfusions of anti-coagulated blood. Another series of experiments involved long-term cannulation of the iliac veins of mice bearing i.m. T241 fibrosarcomas which were locally perfused with physiological medium (Liotta *et al.*, 1974). However, as the artefactual effects of prolonged transfusions or perfusions on circulatory dynamics and cancer cell shedding are not known, they are best avoided as in the present study. A third, more recent series of experiments (Schirrmacher & Waller, 1982) monitored lymphoma cells shed from s.c. tumours. Blood samples were taken from the retro-orbital sinus but such samples contain only those cancer cells which survive passage through the lungs, the first major organ encountered after their release from the primary tumour. The proportion of malignant cells which do not survive this passage, or undergo changes in doing so, can reach over 90% of the dose initially delivered (Weiss, 1980). Previous estimates of the numbers of cancer cells in the blood of mice bearing Lewis lung carcinomas have been reported (James & Salsbury, 1974; Salsbury *et al.*, 1974) but in these earlier studies blood samples from subclavian veins were pooled, cancer cells were counted in buffy coat smears with no indication of the efficiency of the collection methods and the numbers of cancer cells were expressed "per sample" rather than per unit volume so that, at best, only semi-quantitative estimates were obtained. The scope of the present studies is also more extensive than in previous reports since the rates at which cancer cells are naturally shed into the circulation are compared not only with the incidence of spontaneous metastases but also with the behaviour of the cancer cells during intermediate stages of arrest and retention in the organ of secondary growth.

The numbers of cancer cells in the circulation at any one time depend not only on the detachment of cells from the primary tumour (Weiss & Ward, 1983) but also their accessibility to the vasculature and the rates at which they are removed from the circulation. Each of these determinants represents a complex process which may be independently variable in different tumour types. Thus, the extreme vascularity of the Lewis lung carcinomas reported previously (Salsbury *et al.*, 1974) and also seen here, is not observed in the B16 tumours which have comparatively few, small, blood vessels. Therefore, no attempt will be made to compare the behaviour of the tumours on a mechanistic basis; the data refer to numbers of circulating cancer cells regardless of their mode of entry into the blood-stream. The purpose of the present study is restricted to relating, in two distinct tumour types, the numbers of cancer cells released into the blood with the size of the primary tumours generating them and to seek correlation between the numbers of circulating cancer cells and metastatic status, as summarized in Table III.

**Table III** Quantitative relationships between circulating cancer cells and post-dissemination stages of metastasis

| | *Lewis carcinoma* | *B16 melanoma* |
|---|---|---|
| Pulmonary metastases[a] | 88 | 0 |
| Circulating cancer cells[b]: | | |
| total | $1 \times 10^8$ | $2.4 \times 10^7$ |
| clumps | $5 \times 10^6$ | $2.5 \times 10^5$ |
| with leukocytes | $1.9 \times 10^7$ | $2.2 \times 10^6$ |
| Pulmonary arrest | 87.3% | 83% |
| Pulmonary retention (after 24 h) | 0.5% | 1.3% |
| Pulmonary growth (lung colonies) | 0.5 | 6 |

[a]Median at 21 days of primary tumour growth.
[b]Median no. cells shed throughout tumour growth based on a sampling time of 1 min.

Of the 6 parameters measured, 3 were numerically correlated with the incidence of spontaneous metastases. Whatever the mechanism of entry into the blood-stream, the greater numbers of cancer cells found in animals bearing Lewis carcinomas were consistent with the greater frequency of metastasis from this tumour on at least a semi-quantitative basis. However, the numbers of circulating cancer cells in both the Lewis and B16 tumours were orders of magnitude greater than the numbers of spontaneous metastases from either tumour, which reinforces the concept that metastasis is an inefficient process in terms of cancer cell economics (Weiss, 1980, 1982). Pulmonary retention experiments with radiolabelled cells indicated that the death of cancer cells shortly after their arrest in the lung vasculature is probably a major contributor to this inefficiency. However, it remains possible that cells shed spontaneously from primary tumours may not be viable or tumorigenic before entry into the pulmonary vasculature. In the present experiments an indication of the viability of circulating cancer cells has been expressed in terms of dye exclusion. Extensive studies combined with bioassays are obviously required to characterize the tumorigenic potential of circulating cancer cells. These experiments have been made and the results show that almost all circulating cells are potentially tumorigenic but these studies will be reported in depth elsewhere (Mayhew & Glaves, in preparation) However, for the present, the results will be simply interpreted to indicate that the majority of circulating cells were viable.

Whilst variations in the rates of shedding from individual tumours caution against averaging procedures, these fluctuations do illustrate the complexity of the processes involved in metastasis. These fluctuations were not artefactual since they were not observed in recoveries of the artificial mixtures of known numbers of cancer cells and normal blood. They also suggest that the release of cancer cells into the circulation probably occurs sporadically so that samples taken over short periods will necessarily contain variable numbers of cells. Recent studies with a mouse lymphoma (Schirrmacher & Waller, 1982) showed similar fluctuations in the numbers of cancer cells isolated from individual mice at different stages of tumour growth.

In the experiments to evaluate cell loss during the isolation procedures artificial mixtures of enzyme-dispersed cancer cells and blood were used. It is well-known that proteolytic digestion alters cell surfaces and it has been shown that at least some types of cancer cells lose appreciable dry mass as a result of trypsin treatment (Weiss, 1958). It is therefore possible that separation experiments involving enzyme-treated cells may be inappropriate to recovery experiments made on the venous blood of tumour-bearing mice. However, local enzyme activity is one of the factors which contribute to the separation of cancer cells from the primary tumour (Poole, 1973; Weiss & Ward, 1983) and the results are not necessarily artefactual.

Tumour cell aggregates have previously been shown to generate disproportionately more lung colonies than equivalent numbers of single cells after tail-vein injections (Liotta *et al.*, 1976; Fidler, 1973) and the yield of spontaneous metastases was also related to the release of clumps from T241 fibrosarcomas (Liotta *et al.*, 1974). It was therefore of great interest that the release of cancer clumps was the parameter quantitatively most related to overall rates of spontaneous metastasis from the 2 tumours in the present study. In experiments with both types of cancer cells there was no disproportionate loss of clumps during isolation procedures and, therefore, these results do not appear to be artefactual on this account. Since pulmonary retention experiments showed that capillaries are capable of arresting as many as 83–87% of B16 or Lewis cells after injection of single cell suspensions, the presence of clumps would increase trapping efficiency to only a limited extent. It is more likely that emboli containing more than one cell have survival advantage during subsequent stages of metastasis. Possibly greater proportions of clumped than single cells withstand those host specific immune factors (Fidler *et al.*, 1977; Weiss & Glaves, 1976) and non-specific defence factors (Glaves, 1980; Riccardi *et al.*, 1979) involved in clearance of arrested cancer cells from the lungs.

In this study the numbers of circulating tumour cells with adherent lymphocytes was the second most quantitatively correlated factor related to metastatic potential. These observations were especially interesting since previous reports have indicated that lymphocyte attachment to B16 melanoma variants following *in vitro* incubation variably affected their lung colonization potential depending upon the numbers and tumour-sensitization status of the attached lymphocytes (Fidler, 1975). Also, the metastatic T241 fibrosarcoma spontaneously shed cells in association with leukocytes (Liotta *et al.*, 1974) but, as in the present study, the mechanism of the contribution of adherent leukocytes to the success or failure of the metastatic process could not be determined.

The pulmonary arrest patterns of radiolabelled cells injected into tail veins were similar in both B16 melanoma and Lewis lung carcinoma systems. However, as shown by the present and previous observations (Fidler, 1970; Glaves, 1980; Weiss, 1980), the numbers of tumour cells delivered to, and initially arrested in the lungs is less important than the numbers which are actually retained there since the majority of arrested cells are rapidly cleared from the pulmonary vasculature by processes leading to their death or relocalization to other organs. A differential retention pattern was observed following i.v. injection, as more radiolabelled B16 melanoma than Lewis carcinoma cells were retained in the lungs, in addition more pulmonary colonies developed following i.v. injection of non-radiolabelled melanoma than carcinoma cells. Thus, according to these experimental models, the behaviour of the two tumours during the post-dissemination phases of metastasis is not directly related to their relative *spontaneous* metastatic potentials, which is similar to the results of previous work with a series of B16 melanoma variants (Weiss *et al.*, 1982).

Although the diagnosis of circulating cancer cells in Man has presented problems in the past (eg. McGrew, 1965) part of this was due to deterioration in specimens with time. Thus, the longer the delay before the specimen was fixed, the more frequently were cancer cells diagnosed! In addition, megakaryocytes, particularly in degenerative states, were also often confused with malignant cells. In the present investigation the blood specimens were processed and fixed within 120 min of withdrawal from tumour-bearers. Also, duplicate samples were prepared, one was treated with Wright's stain to distinguish haematogenous elements and the other with Papanicolou's stain which is the classical stain used for the diagnosis of malignant cells. Cancer cells were diagnosed on the basis of those multiple morphological criteria previously used for the identification of cancer cells shed from T241 fibrosarcomas in mice (Liotta *et al.*, 1974) and described earlier. Also in contrast to previous reports, many of which were essentially concerned with diagnosis, identification was limited to the recognition of two known types of cancer cells with which the author has considerable experience so that identification presented little problem in this respect (Figure 3).

**Figure 3** Circulating cancer cells compared with bone-marrow derived cells (a) clump of carcinoma cells with attached lymphocyte (b) large clump of carcinoma cells showing moulded configuration (c) clump of two carcinoma cells with 6 closely opposed lymphocytes (d) bone marrow aspirate, note central megakaryocyte (e) circulating megakaryocyte, from melanoma-bearer (f) circulating megakaryocyte nucleus denuded of cytoplasm, from melanoma-bearer. The pores of the polycarbonate filters can be seen in the background. Magnification ×250, Papanicolou.

In conclusion, these studies provide quantitative data on spontaneous cancer cell input into the metastatic cascade. They also identify particular points in the cascade at which two tumours with different metastatic frequencies diverge in their behaviour. However, it is the behaviour of B16 and Lewis cells before they reach the organ of arrest and potential secondary growth which is more directly related to their overall rates of spontaneous metastasis. Commonly used experimental models of metastasis involve i.v. injection of cancer cells which bypasses earlier stages of the process, yet these earlier stages seem to be major contributors to the successful development of metastases. Indeed, it appears that the low lung colonization potential of Lewis carcinoms cells is compensated for during spontaneous metastasis by the high numbers of single and clumped cells shed into the circulation from the primary tumour.

The excellent technical assistance of Deborah Ketch is appreciated and the helpful advice and encouragement of Dr. L. Weiss is acknowledged. Funding for this research was provided by Grant No. CA 28362 and partly by Core Grant No. 5P30 CA 16056 from the National Institute of Health.

### References

BISHOP, C.J., SHERIDAN, J.W. & DONALD, K.J. (1981). The effect of $^{125}$I-5-iododeoxyuridine labelling on murine tumour cells. *Br. J. Exp. Pathol.*, **62**, 22.

BUTLER, T.P. & GULLINO, P.M. (1975). Quantitation of cell shedding into efferent blood of mammary adenocarcinoma. *Cancer Res.*, **35**, 512.

FIDLER, I.J. (1970). Metastasis: quantitative analysis of distribution and fate of tumour emboli labeled with $^{125}$I-5-iodo-2′-deoxyuridine. *J. Natl Cancer Inst.*, **45**, 773.

FIDLER, I.J. (1973). The relationship of embolic homogeneity, number size, and viability to the incidence of experimental metastasis. *Eur. J. Cancer*, **9**, 223.

FIDLER, I.J. (1975). Biological behavior of malignant melanoma cells correlated to their survival *in vivo*. *Cancer Res.*, **35**, 218.

FIDLER, I.J., GERSTEN, D.M. & RIGGS, C.W. (1977). Relationship of host immune status to tumor cell arrest, distribution, and the survival in experimental metastasis. *Cancer*, **40**, 46.

GLAVES, D. (1980). Metastasis: reticuloendothelial system and organ retention of disseminated malignant cells. *Int. J. Cancer*, **26**, 115.

HOFER, K.G., PRENSKY, W. & HUGHES, W.L. (1969). Death and metastatic distribution of tumor cells in mice monitored with $^{125}$I-Iododeoxyuridine. *J. Natl Cancer Inst.*, **43**, 763.

JAMES, S.E. & SALSBURY, A.J. (1974). Facilitation of metastases by antithymocyte globulin. *Cancer Res.*, **34**, 367.

LIOTTA, L.A., KLEINERMAN, J. & SAIDEL, G.M. (1974). Quantitative relationships of intravascular tumor cells, tumor vessels, and pulmonary metastases following tumor implantation. *Cancer Res.*, **34**, 997.

LIOTTA, L.A., KLEINERMAN, J. & SAIDEL, G.M. (1976). The significance of hematogenous tumor cell clumps in the metastatic process. *Cancer Res.*, **36**, 889.

McGREW, E.A. (1965). Criteria for the recognition of malignant cells in circulating blood. *Acta Cytol.*, **9**, 58.

POOLE, A.R. (1973). Tumor lysosomal enzymes and invasive growth. In: *Lysosomes in Biology and Pathology* (Ed. Dingle) Vol. 3. p. 303 North-Holland: Elsevier.

PROCTOR, J.W., MASTROMATTEO, W.P., ANTOS, M. & HEDDERSON, E.D. (1979). Variations in the level of haematogenous antitumour immunity during progressive tumour growth and spontaneous blood-borne metastatic spread. *Oncology*, **36**, 49.

RICCARDI, C., PUCCETTI, P., SANTONI, A. & HERBERMAN, R.B. (1979). Rapid *in vivo* assay of mouse natural killer cell activity. *J. Natl Cancer Inst.*, **63**, 1041.

SALSBURY, A.J., BURRAGE, K. & HELLMAN, K. (1974). Histological analysis of the antimetastatic effect of (±)-1,2-bis (3,5-dioxopiperazin-1-yl) propane. *Lances Res.*, **34**, 843.

SCHIRRMACHER, V. & WALLER, C.A. (1982). Quantitative determination of disseminated tumor cells by $^{3}$H-thymidine incorporation *in vitro* and by agar colony formation. *Cancer Res.*, **42**, 660.

WEISS, L. (1958). The effects of trypsin on the size, viability, and dry mass of sarcoma 37 cells. *Exp. Cell Res.*, **14**, 80.

WEISS, L. (1980). Cancer cell traffic from the lungs to the liver: an example of metastatic inefficiency. *Int. J. Cancer*, **25**, 385.

WEISS, L. (1982). Metastatic inefficiency. In: *Liver Metastasis* (Eds. Weiss & Gilbert) p. 126 Boston: G.K. Hall & Co.

WEISS, L. & GLAVES, D. (1976). The immunospecificity of altered arrest patterns of circulating cancer cells in tumor-bearing mice. *Int. J. Cancer*, **18**, 774.

WEISS, L. & WARD, P. (1983). Cell detachment and metastasis. *Cancer Metastasis Rev.* (in press).

WEISS, L., MAYHEW, E., RAPP, D.G. & HOLMES, J. (1982). Metastatic inefficiency in mice bearing B16 melanomas. *Br. J. Cancer*, **45**, 44.